# Effects of Different Cold Preservation Solutions on the Functions of Cultured Isolated Human Hepatocytes

**Published:** 2020

**Authors:** M. Hossein Aghdaie, N. Azarpira, A. Shamsaeefar, N. Motazedian, M. Kaviani, E. Esfandiari, S. Golbabapour, S. Nikeghbalian, K. Kazemi, H. Salahi, S. A. Malek-Hosseini, B. Geramizadeh

**Affiliations:** 1 Transplant Research Center, Shiraz University of Medical Sciences; 2 Department of Hepatobiliary Surgery, Shiraz University of Medical Sciences; 3 Department of Pathology, Shiraz University of Medical Sciences

**Keywords:** Liver transplantation, Cryopreservation, Hepatocytes

## Abstract

**Background:**

Hepatocyte transplantation using isolated human hepatocytes is an alternative source that can be used for the treatment of metabolic diseases and acute liver failure as a time bridge to liver transplantation. These cells can also be used for bioartificial liver systems and *in vitro* study of drug toxicity.

**Objective:**

To determine which cold preservation solution is better maintain the liver function.

**Methods:**

We prepared 4 cold preservation solutions made of different combination of antioxidants, chelating, membrane protective, and anti-apoptotic agents as well as inhibitor of cyclophilin D. For hepatocyte isolation, we used livers obtained from unused deceased donor livers and the liver of patients with Crigler-Najjar syndrome who were candidates of partial liver transplantation. After culture and cold preservation, the level of albumin, and urea production were measured as indices of liver functionality.

**Results:**

We found that albumin production significantly decreased after cold preservation in solution 1. There was no significant difference in urea production after cold preservation in solution 1 compared with control 24 h. No significant differences in albumin production were found after cold storage in solution 2 and solution 4 compared with control 24 h. Urea production significantly decreased after cold storage in solutions 2 and 4 compared with control 24 h. As a whole albumin and urea production were significantly decreased after cold preservation. Although albumin and urea production were decreased after cold preservation, but the results of albumin production of two solutions were not significantly different from that of the control group (p=0.109 and 0.951).

**Conclusion:**

Cold preservation of cultured human hepatocytes in solution 2 and solution 4 could maintain the function of albumin production better than other cold preservation solutions in our experiments; solution 1 was more effective on urea production of cultured human hepatocytes at 4 °C for 24 h. To determine if these hepatocytes are suitable candidates for transplantation, further studies should be performed.

## INTRODUCTION

Orthotropic liver transplantation (OLT) is the standard treatment for end-stage liver diseases. However, for shortage of suitable transplantable organs, alternative sources such as isolated hepatocytes have been investigated for many years for the treatment of acute liver failure as a time bridge until the time of liver transplantation. Hepatocyte transplantation has also been used as a treatment modality for metabolic disorders [1, 2]. A large number of cells are necessary for the purpose of cell transplantation in acute liver failure and congenital liver diseases. However, there is limited number of livers for isolation of hepatocytes. Therefore, storage of isolated hepatocytes is very important, especially when the number of isolated cells is high. Isolated hepatocytes could be either cryopreserved or cold stored [3].

By cryopreservation, high quantity of cells could be stored for a long time to be accessible at once; however, cold storage causes injuries to the cells and their organelles such as mitochondria. Cold storage also causes decreased ATP levels. The stored cells in cold environment show reduced attachment [4]. Hypothermic storage (2–8 °C) keeps the cells accessible in the case of programmed biotherapy [3, 5]. 

In this study, we used cultured human hepatocytes to evaluate the effect of different preservation solutions on the maintenance of hepatocytes’ functionality [9-12].

## MATERIALS AND METHODS

In this study we used the tissue of unused deceased-donor liver for hepatocyte isolation as well as the liver of patients with Crigler-Najjar syndrome who were candidates for partial liver transplantation from either their parents or deceased donor. The samples were obtained after informed written consents and approval were taken from the local Ethics Committee of Shiraz University of Medical Sciences.

Hepatocytes were isolated from five liver donors—four were explanted livers of patients with Crigler-Najjar syndrome age between 1.3 and 8 years. The other one was taken from a deceased-donor liver tissue. Characteristics of the liver tissues are shown in Table 1. After harvesting by transplant surgeons and clinicopathological evaluation of the donor liver tissues by an expert pathologist, the livers were sent to our research lab. Immediately after tissue transportation to the lab, the right lobe of the liver or segments II and III were sectioned and flushed with cold 0.9% sodium chloride and 5% dextrose (1:1 v/v). Afterwards, the tissues were flushed with and then kept in 4 °C UW solution (SPS-1, USA) overnight.

**Table 1:** Characteristics of liver tissue donors used for isolation of hepatocytes

No	Sex	Age (yrs)	Cold ischemic time (hrs)	Yield of cells (×10^6^)	Percentage of steatosis	Pathological problem
1	F	36	44	0.79	20% microvesicular, 10% macrovesicular	Atherosclerosis
2	M	8	22	6.46	—	Crigler Najjar
3	M	1.3	22	14.12	—	Crigler Najjar
4	M	2	12	15.44	—	Crigler Najjar
5	F	1.7	23	8.06	—	Crigler Najjar

Isolation of Human Hepatocytes and Culture

Hepatocytes were isolated by collagenase perfusion method as described earlier [6]. Large hepatic vessels were cannulated by intravenous cannulae (12–18G, CalMed Laboratories, Medi Mark Europe) through which the liver tissue was flushed with three perfusion solutions. The cannulated tissue and perfusion tubes were kept in a sterile organ bag (3M Healthcare) and put in water bath at 37 °C.

The perfusion steps were done by using the peristaltic pump heads (Cole-Parmer) with a flow rate of 60–80 mL/min. The first perfusion solution included calcium and magnesium-free Hanks’ Balanced Salt Solution (HBSS; Shelmax, H7015) containing 0.5 mM/L ethylene glycol tetra acetic acid (EGTA; Sigma cat# E4378) and 5 mM N-acetyl cysteine (Sigma-Aldrich, cat#A9165).

The second solution was HBSS without EGTA. Afterwards, the digestion of tissue was done with 0.5 g/L collagenase P in Williams E medium without phenol red (Lonza, cat#BE02-019F) and 50 mg/L DNase (DN25, Sigma-Aldrich, CAS #9003-98-9), with the exception of tissues of younger donors that half of the amounts of collagenase and DNase were necessary. The digestion took about 7–30 minutes during which period the solution was circulated. The digested tissue was mechanically disrupted in cold high glucose DMEM (Biosera cat#1110) containing 5000 U/mL heparin to prevent cell clumping and filtered through sterile gauze and centrifuged at 50 g at 4 °C for 5 min.

After washing the hepatocytes with high-glucose DMEM thrice, they were suspended in Williams E medium (WEM) supplemented with 10% FBS (Gibco, cat#10270-106), 32U/L insulin (Alborz Daru), 0.9 mg/L dexamethasone (Alborz Daru), penicillin-streptomycin (Bioidex, B11036), L-glutamine (Gibco, cat#35050-038) and 1 M HEPES (Lonza, cat#BE17-737E). For determination of viability and number of hepatocytes, trypan blue (Sigma-Aldrich) exclusion test was used. Then, equal volume of 0.4% w/v trypan blue (Sigma, T6146) in phosphate buffered saline (PBS) and sample of cell suspension were mixed and viability of hepatocytes was expressed as percentage of viable cells of the total the cells.

Freshly isolated viable hepatocytes (5×10^5^ cells) were seeded in each well of collagen type I-coated (BD Biocoat, cat#356408 BD Bioscience) 24-well plates in WEM medium supplemented as mentioned above in triplicate. Four plates were allocated to cold storage experiments. The plates were kept at 37 °C, 96% humidity, 95% air and 5% CO_2_ overnight.

Cold Preservation Solutions 

UW is a standard organ preservation solution which can also be used for cold preservation of hepatocytes [7-9]. We used UW as the main solution for cold storage and other solutions were prepared by adding different materials and solutions to UW including antioxidants such as α-lipoic acid (Sigma-Aldrich, cat#T1395), vitamin E (Sigma-Aldrich, cat#T3251), human serum albumin (HSA) (20%-CSL, Behring), PEG 35 kDa (Sigma-Aldrich, cat#, 94646), UDCA (Sigma-Aldrich, U5127) as an antiapoptotic agent, deferoxamine (Sigma-Aldrich, D9533) as an Iron chelating agent, and cyclosporine A (Sigma, 30024) as an inhibitor of CyP-D. Some of the chemicals (vitamin E, UDCA, α-lipoic acid, and CsA) were dissolved in ethanol to make a total concentration below 4% by volume [10]. The pH of cold preservation solutions were adjusted to 7.2–7.4. The composition of different cold preservation solutions is shown in Table 2.

**Table 2:** Composition of Cold Preservation Solutions

Solution	UW	Vitamin E (100 µM/L)	Deferoxamine (1 mM/L)	HSA (5%)	PEG (1%)	CsA (1 µM/L)	α-Lipoic acid (5 mM/L)	UDCA (0.5 mM/L)
1	+	–	–	–	–		–	–
2	+	+	+	+	+	+	–	–
3	+	–	–	–	–	–	+	+
4	+	+	+	+	+	+	+	+
WEM	–	–	–	–	–	–	–	–

HSA: Human serum albumin; PEG: Polyethylene glycol; CsA: Cyclosporine A; UW: University of Wisconsin; UDCA: Ursodeoxycholic acid; WEM: 500 mL Williams E medium supplemented with 32 U/L insulin, 0.9 mg/L dexamethasone, penicillin-streptomycin (5 mL), L-glutamine (5 mL), and 1 M HEPES (5 mL)

Cold Storage Experiments

**Figure 1:**
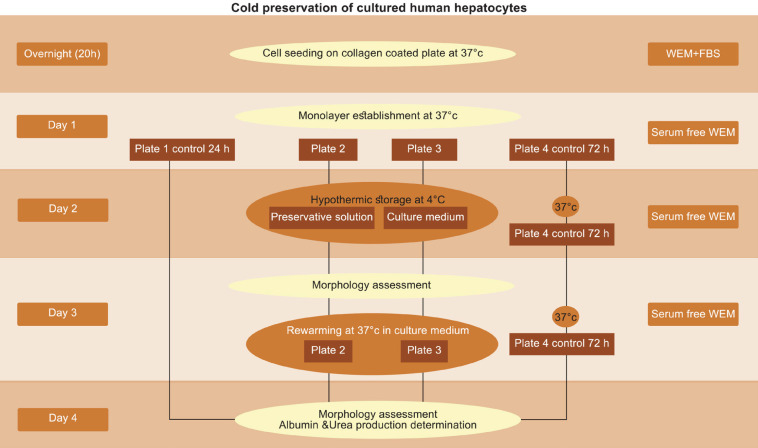
Flowchart of experimental design. Hepatocytes were cultured in 24-well collagen-coated plates at 37 °C for 24 hrs; then cold preserved at 4 °C for 24 hrs. After cold preservation hepatocytes were cultured in serum free WEM at 37 °C for 24 hrs. Then, albumin and urea production were determined. Morphology assessment was performed after 24 hrs and 72 hrs as the controls, immediately after cold preservation and rewarming the cells 24 hrs at 37 °C

After 20 hours (overnight attachment) (Fig 1), the medium was removed. After washing the cells with WEM medium without FBS (serum free WEM), they were cultured for 24 hrs in the same medium. After 24 hrs (1^st^ day), plate 1, which was considered “normothermic control or 24 h control” was evaluated morphologically. The supernatants of the cells of this plate were removed and centrifuged at 1500 RPM at 4 °C for 10 min and stored at -80 °C for further analysis. Due to normalization of the values in hepatocyte supernatants for the determination of albumin and urea production, cell pellet of each well were lysed by RIPA buffer according to the manufacturer (Sigma-Aldrich, cat# R0278) to solubilize the protein from the cells; subsequently, the protein content of the cells was determined by Bradford method (Bio-Rad Protein Assay, cat# 500-0002,500-0006). The cells of the other plates were washed with serum free WEM (37 °C). Plate 2 was allocated to cold preservation solution treatment; before adding different cold preservation solutions, the cells were preincubated with WEM medium supplemented with the same substances as cold preservation solutions (except of PEG) at 37 °C for 60 min to enhance the absorbance of the substances through cell membrane. Afterwards, supernatants of the cells were removed and different cold preservation solutions were added to the wells. Plate 3 was allocated to cold preservation of hepatocytes in culture medium (serum free WEM); some of the wells were allocated to WEM with ethanol as solvent control. Plates 2 and 3 were kept at 4 °C for 24 hrs undisturbed (2^nd^ day). After 24 hrs cold storage, the cells of plates 2 and 3 were washed with culture medium and serum free WEM 37 °C was added to the wells; the plates were then kept in incubator as mentioned above for 24 hrs (3^rd^ day). At the 4^th^ day of the experiment, the supernatants of the wells of all plates were removed and centrifuged as mentioned and stored at -80 °C. The cell pellets of all wells were lysed and their protein was extracted for the determination of total protein content. Plate 4 was kept in incubator at 37 °C throughout the experiment and the media of the cells were changed at each step of the experiment. This plate was considered “control of 72 h” incubation at 37 °C.

Evaluation of Cellular Morphology

Morphology of the cells was assessed by inverted phase contrast microscope (Olympus CKX 41). 

Quantification of Albumin and Urea Production and Secretion 

Human albumin in the supernatants of cultured hepatocytes was measured by a sandwich ELISA method according to the manufacturer’s instructions (Bethyl Laboratories, Inc, USA). The concentration of albumin was calculated by a program through the web site elisaanalysis.com/app by establishing a 4-parameter, logistic regression curve to analyze the data. Urea production and secretion by hepatocytes was assessed by the quantitative colorimetric urea determination kit (Quantichrom urea assay kit, DIUR-500 BioAssay systems). The values of albumin and urea in supernatants of hepatocytes were normalized with the total protein content, which was extracted from the cells of the same well and quantified with Bradford method.

Statistical Analysis

SPSS^®^ for Windows^®^ ver 16 was used for data analysis. The experiments of culture of isolated hepatocytes and assessment of their protein content were done in triplicate. Measurement of albumin and urea was performed in duplicate. Due to the small sample size, data analysis was done using Kruskal-Wallis test. Post hoc tests were used to test pairwise comparisons. A p value <0.05 was considered statistically significant.

## RESULTS

In our experiments, primary hepatocytes were isolated from four patients with Crigler-Najjar syndrome. The livers looked healthy. Their isolated hepatocytes had high viability and yield and formed confluent monolayer on collagen-coated plates. One of our patients had atherosclerosis and long cold ischemic time. Therefore, the yield of the cells was very low (Table 1). 

Morphology of Hepatocytes after Cold Preservation and Rewarming

**Figure 2:**
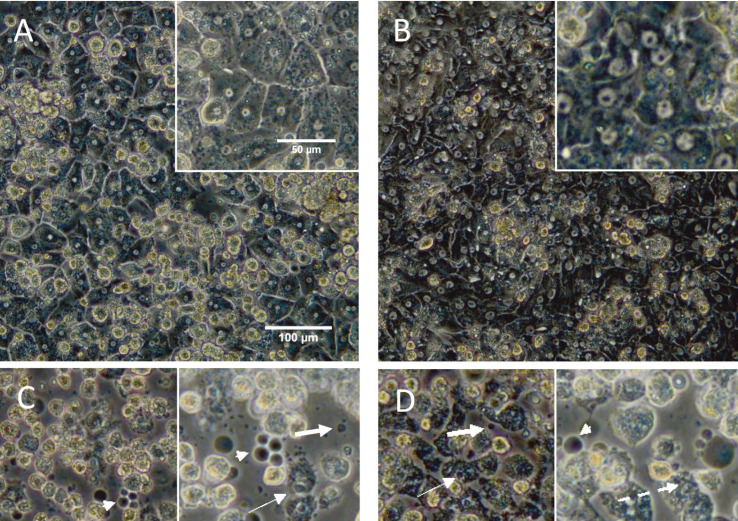
Morphology of cultured human hepatocytes after 24 hrs of hypothermic storage (4 °C) and rewarming. Cultured cells stored at 4 °C in serum free Williams E medium (WEM) (C, D) and University of Wisconsin (UW) solution (E, F) and rewarmed in serum free WEM for 24 hrs at 37 °C and 5% CO_2_. Morphological assessment was performed by phase contrast microscopy immediately after cold storage (C, E) and after rewarming (D, F). Control cells incubated for 24 hrs (A) and 72 hrs (B) at 37 °C and 5% CO_2_. Bleb formation (arrowhead), apoptotic bodies (thick arrows), nuclei condensation (thin arrows) and vacuolization (dashed arrow) are shown

To assess the morphology of cold preserved samples after 24 hrs, supernatants of the cells were removed; the cells were then washed with serum free WEM. Then, fresh medium was added to the cells and their morphology was checked by inverted phase-contrast microscope. Furthermore, after 24 hrs of rewarming at 37 °C, supernatants of the cells were removed and fresh medium was added; morphology of these cells was also evaluated. The results showed that hepatocytes cultured at 37 °C for 24 hrs (normothermic plate) displayed normal monolayer appearance with polygonal shape with one or more nuclei, granular cytoplasm and formation of bile canaliculi-like structures which is indicated as light area between cells (Fig 2A). The hepatocytes of 72-hr control group also showed normal morphology (Fig 2B). Cold preserved hepatocytes in culture medium WEM at 4 °C for 24 hrs displayed cold-induced injuries such as blebbing of the cells, condensation of nuclei and formation of apoptotic bodies immediately after cold storage (Fig 2C). After 24 hrs of rewarming, vacuolization was seen in the cytoplasm too (Fig 2D).

**Figure 3:**
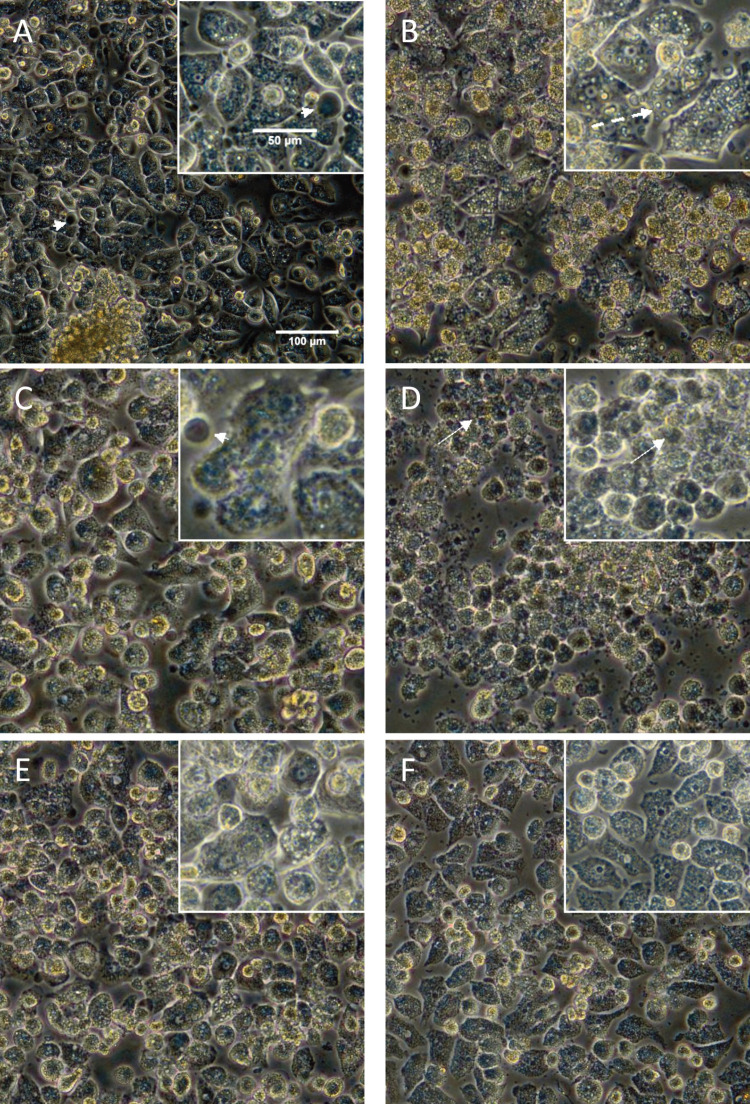
Morphology of cultured human hepatocytes after 24 hrs of hypothermic storage (4 °C) and rewarming. Cultured cells stored at 4 °C in different cold preservation solutions and rewarmed in serum free Williams E medium for 24 hrs at 37 °C and 5% CO_2_. Solution 2 (A, B), solution 3 (C, D), solution 4 (E, F): morphological assessment was performed by phase-contrast microscopy immediately after cold storage (A, C, E) and after rewarming (B, D, F). Bleb formation (arrowhead), nuclei condensation (thin arrows) and vacuolization (dashed arrow) are shown

Hepatocytes which have been preserved in UW solution (solution 1) retained a morphology very similar to that of cells stored in normothermic condition; however, in comparison with normothermic control plate, cell detachment was seen (Fig 2E). After 24 hrs of rewarming, vacuolization and a few blebs were seen (Fig 2F). To improve hypothermic preservative efficiency of solution 1, deferoxamine was added to the solution to protect the cells against cold-induced injury, which would be triggered by cellular iron ions; PEG was used to stabilize cell membrane of the cells during temperature changes. Vitamin E and HSA were added as antioxidants and CsA was used as CyP-D inhibitor. When hepatocytes cold preserved in this solution (solution 2), the polygonal shape of the cells maintained; most of the nuclei were intact; however, the cell granularity increased in comparison with the control cells (Fig 3A). Although, vacuolization was seen in the cells after rewarming, most of the nuclei were remained intact (Fig 3B). 

Due to antiapoptotic effects of UDCA, which have been studied in isolated human hepatocytes [11], and regarding antioxidant function of α-lipoic acid, these substances were added to solution 1 to prepare the solution 3 to investigate their effects on the protection of hepatocytes during cold preservation. Cold preservation of hepatocytes in solution 3 caused changes in the polygonal shape of the cells and nuclei in comparison with the control hepatocytes, which were evident immediately after cold storage (Fig 3C). After rewarming the cells at 37 °C, most of the cells were retracted and their nuclei were condensed (Fig 3D).

Solution 4 had the composition of solutions 2 and 3 to study the additive effects of their substances on the protection of the cells against cold-induced changes. In this solution, some of the cells were retracted; most of the nuclei seemed to be intact immediately after cold storage (Fig 3E). Although cell granularity increased in comparison with the control hepatocytes after rewarming, the shape of the cells was polygonal (Fig 3F).

Effects of Preservation Solutions on Albumin Production by Cultured Human Hepatocytes Compared with the Control Hepatocytes 24 h and 72 h

**Figure 4:**
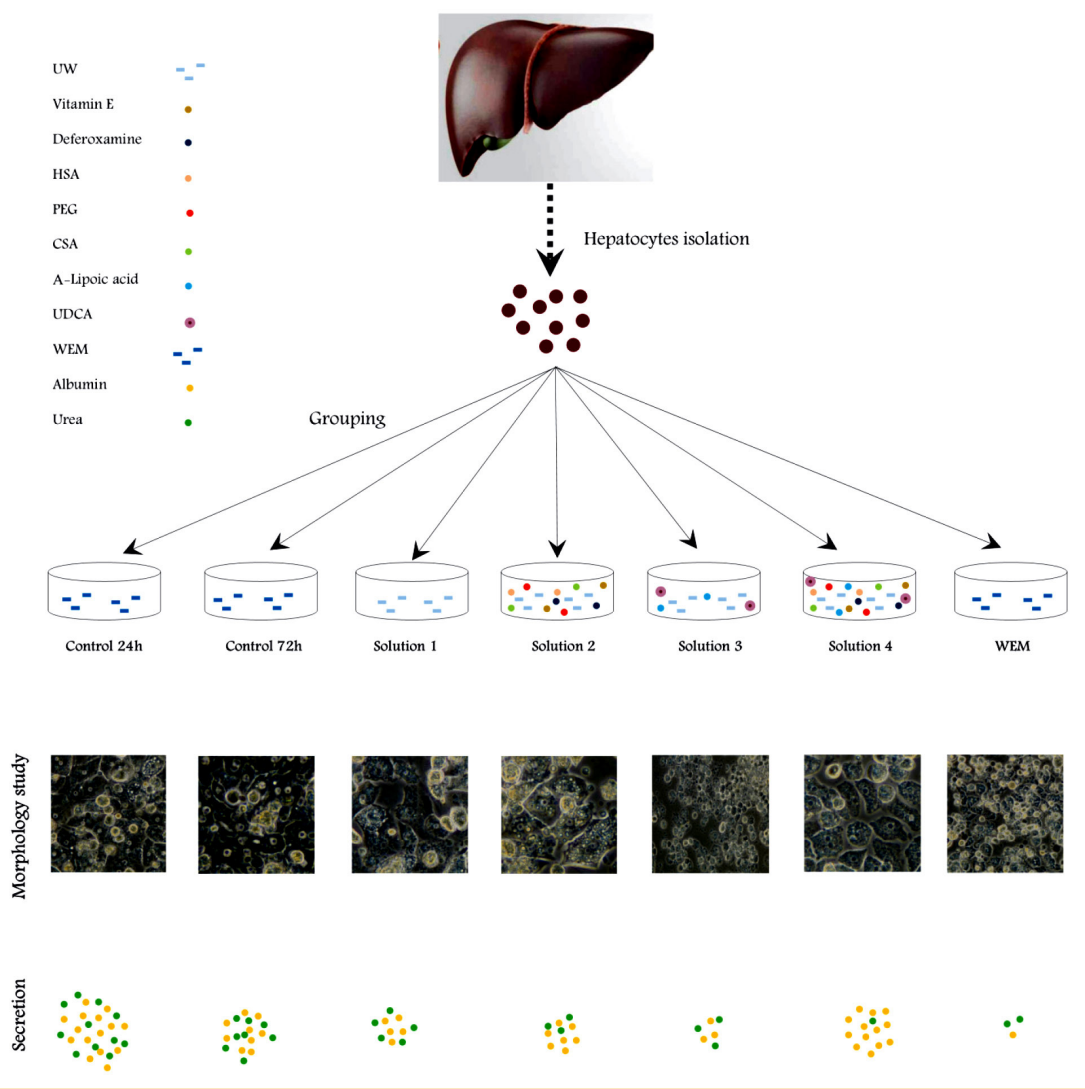
Schematic diagram of experimental steps showing the relationship between different cold storage solutions and the amount of albumin and urea production. After hepatocyte isolation, they were cultured and allocated to different cold storage groups. After 24 hrs of storage at 4 °C, they were rewarmed at 37 °C and their albumin and urea production were measured

To evaluate the changes in functional efficiency of cultured human hepatocytes after cold preservation, the amount of albumin and urea produced by different groups of cold preserved cultured hepatocytes were determined after 24 hrs of rewarming at 37 °C and compared with control groups. The results are shown in Table 3 and Figure 4. There was a significant (p<0.001) difference in mean albumin production between groups studied. Therefore, we conducted post hoc tests to test pairwise comparisons. 

**Table 3:** Comparison of albumin production in cultured isolated human hepatocytes in different preservation solutions with control 24 h and 72 h hepatocytes

Studied group	Albumin (ng/mL), Mean±SD	Mean rank	p value (*vs*. Control 24 h)	p value (*vs*. Control 72 h)
Control 24 h	424.73±205.21	29.20		
Control 72 h	253.55±76.72	23.60	0.388	
Solution 1	115.49±46.00	13.20	0.014	0.109
Solution 2	191.20±53.50	18.80	0.109	0.460
Solution 3	59.86±31.62	7.80	0.001	0.015
Solution 4	369.79±93.79	29.60	0.951	0.355
WEM	27.18±17.94	3.80	0.001	0.002

Solution 1: University of Wisconsin (UW); Solution 2: UW+(vitamin E 100 µM/L, deferoxamine 1 mM/L, human serum albumin (HSA) 5%, polyethylene glycol (PEG) 1%, and cyclosporine (CsA) 1 µM/L); Solution 3: UW+(α-lipoic acid 5 mM/L, ursodeoxycholic acid (UDCA) 0.5 mM/L); Solution 4: substances of solutions 2 and 3; WEM: serum free Williams E medium. Kruskal-Wallis test and pairwise comparison were performed (n=5).

No significant difference was observed after cold storage in solutions 2 and 4 compared with control 24 h and72 h.

Effect of Preservation Solutions on Urea Production by Cultured Human Hepatocytes Compared with control Hepatocytes 24 h and72 h

The results showed a significant (p=0.008) difference in mean urea production among groups; post hoc tests were carried out to test pairwise comparisons (Table 4). There was no significant difference in urea production after cold preservation in solution 1 compared with control 24 h and 72 h.

**Table 4:** Comparison of urea production in cultured isolated human hepatocytes in different preservation solutions with control 24 h and 72 h hepatocytes

Studied group	Urea(mg/dL), Mean±SD	Mean rank	p value (*vs*. Control 24 h)	p value (*vs*. Control 72 h)
Control 24 h	12.04±4.46	31.60		
Control 72 h	5.50±3.75	23.60	0.22	
Solution 1	4.29±3.87	20.20	0.079	0.600
Solution 2	2.41±2.20	15.00	0.010	0.185
Solution 3	2.67±2.75	15.60	0.014	0.217
Solution 4	0.81±0.54	9.20	0.001	0.026
WEM	1.33±1.60	10.80	0.001	0.048

Solution 1: University of Wisconsin (UW); Solution 2: UW+(vitamin E 100 µM/L, deferoxamine 1 mM/L, human serum albumin (HSA) 5%, polyethylene glycol (PEG) 1%, and cyclosporine (CsA) 1 µM/L); Solution 3: UW+(α-lipoic acid 5 mM/L, ursodeoxycholic acid (UDCA) 0.5 mM/L); Solution 4: substances of solutions 2 and 3; WEM: serum free Williams E medium. Kruskal-Wallis test and pairwise comparison were performed (n=5).

## DISCUSSION

Shortage of donor tissue is an important problem for liver transplantation. Hepatocyte therapy is still the most practical alternative to treat end-stage or inborn error of metabolic liver diseases as a time bridge to PLT. Therefore, isolated primary human hepatocytes with good quality are very valuable sources for cell therapy and also for pharmacological studies [3]

Cryofreezing is a useful method to store high quantity of isolated hepatocytes for a long time which are accessible on the time of need. However, due to some disadvantages, such as impairment of plating efficiency, protein synthesis and CYP activity [7], hypothermic storage of hepatocytes at 2–8 °C [3] is of more interest for scheduled biotherapy, transport of hepatocytes from isolation laboratory to transplant center [5], and bioartificial liver devices. Although low temperature is effective in retaining the health of the cells by slowing down the metabolism, it induces some injuries to the cells. Thus, the ultimate goal of researchers is to reduce the extent of cold damages to its lowest possible level. The results of our experiments using WEM without any cold preservative in hypothermic condition were similar to the results of others [12].

Cold preservation of cultured hepatocytes in UW solution resulted in findings similar to those reported by Duret, et al. [5]; they were, nonetheless, different from the results of Stefanovich, et al., in terms of the amount of albumin produced. This might be due to species differences or the quality of donor hepatocytes [13].

Use of solution 2 maintained albumin production efficiently. It is possible that the presence of PEG influenced the stability of the cell membrane and cytoskeletal organization of the cells [13-15]. When we used solution 3, which was composed of UDCA and α-lipoic acid in UW solution, the amount of albumin production was lower than that in the control group. When these substances were added in solution 4, which was composed of the substances of solution 2, the amount of albumin production by cultured hepatocytes was increased in comparison with those observed with other solutions. As a whole, our results showed that cold preservation of cultured human hepatocytes in solutions 2 and 4 could maintain the function of albumin production better than other cold preservation solutions studied in our experiment. The difference between solutions 2 and 4 was the presence of UDCA and α-lipoic acid in solution 4. The possible reason for the higher albumin production in hepatocytes of solution 4 group in comparison to solution 2 might be the binding of UDCA and α-lipoic acid to HSA [16, 17]. It is well known that HSA has high capacity to interact with different endogenous and exogenous compounds and drugs that influence the rate of their delivery and efficacy to liver parenchymal cells [18-20].

Moreover, HSA has a powerful antioxidant capacity due to the existence of many thiol groups in its structure [17]. The binding of UDCA and α-lipoic acid to HSA, as a transporter, may enhance their delivery to the hepatocytes and promote their effects on albumin production. On the other hand, because one of the benefits of PEG is preservation of cellular integrity and stabilization of the cell membrane at low temperature (4 °C) [21], and the iron chelating specificity of deferoxamine, their synergistic effects would result in higher albumin production by cultured hepatocytes which cold preserve in solution 4 compared to other solutions. However, to elucidate these probabilities, further investigations should be performed. Furthermore, we found that there were no significant differences in albumin production of cultured human hepatocytes cold preserved in solutions 1, 2, and 4 in comparison with the control hepatocytes which were incubated 72 hrs at 37 °C.

Another important point of our study was that although our sample size was very small for lack of suitable donor tissue for hepatocyte isolation; however, we found that liver tissues of patients with Crigler-Najjar syndrome (regardless of genetic problem) were invaluable sources for hepatocyte isolation and further experimental studies. Moreover, the amount of necessary enzyme for tissue digestion is half of the amount of adult tissue and the yield of isolated cells are much more than the adult tissue due to tissue softness. In conclusion, we used four cold preservative solutions; solutions 2 and 4 were more effective on albumin production, whereas solution 1 was more effective on urea production of the cultured human hepatocytes. Whether these hepatocytes are suitable candidates for transplantation is a question waiting to be clarified in further studies.
